# Improving the Properties of Magnetite Green Pellets with a Novel Organic Composite Binder

**DOI:** 10.3390/ma15196999

**Published:** 2022-10-09

**Authors:** Shuo Liu, Yuanbo Zhang, Zijian Su, Tao Jiang

**Affiliations:** School of Minerals Processing and Bioengineering, Central South University, Changsha 410083, China

**Keywords:** green pellets, a humic substance-based binder, drying process, water content

## Abstract

A novel composite binder (humic acid modified bentonite, HAMB) and two other binders (bentonite and Modified humic acid, MHA) were used to explore the effects of binders on the properties of magnetite green pellets in this study. The results of green pellet properties and drying tests show that the low doses of a humic substance-based binder can achieve the same effect as high doses of bentonite binder. A humic substance-based binder could be a promising organic binder to replace bentonite. Meanwhile, the influence mechanism of adding different binders on the strength of green pellet was discussed, and the relationship between moisture content in the pellet and the compression strength of three binders was determined. A TG-DSC analysis found that the novel composite binder (HAMB) was not a simple mix of humic acid and bentonite, in which a humic substance could change the structure of bentonite and reduce the thermal stability of bentonite, causing the HAMB composite binder to have a high decomposition temperature.

## 1. Introduction

The binder is an important additive widely applied in pelletizing iron ore concentrates, making iron ore pellets available as feedstocks for blast furnace ironmaking or direct reduction processes [1,2,3]. Bentonite clay, due to its excellent effectiveness and relatively low cost, has been the predominant binder for pellet production since 1950s [4,5,6]. However, as the disadvantages of contaminants (such as silica and alumina) in bentonite clay affect the quality of fired iron ore pellets, many researchers have placed particular emphasis on developing alternative binders to bentonite [5,6,7]. In order to achieve this goal, numerous experiments with organic and inorganic substances, such as starch, carboxymethyl cellulose, polyacrylamide, humic acid, colemanite and sodium metasilicate, etc., were carried out in the laboratory [7,8,9,10,11,12,13,14]. One famous organic binder, called Peridur, has been successfully used as an alternative to bentonite binders in many countries except China, where the grate-rotary kiln process was mainly used to produce pellets. To solve this problem in China, studies on a humic substance-based binder were conducted on the Chinese pelletizing industry. Since the Funa binder was successfully applied in the direct reduction in cold-bonded pellets, the MHA binder has also been successfully applied in vanadium–titanium (V-Ti) magnetite, specularite, and fluxed hematite to produce the oxidized pellets [15,16]. The humic substance-based binder has made a big breakthrough in the pelletizing industry in China [17,18,19].

The properties, preparation methods, behaviors in pelletization and applications of the humic substance-based binder were also extensively studied and discussed. For example, there are two main ingredients in modified humic acid (MHA), named humic acid (HA) and fulvic acid (FA). by the authors’ team clarified the adsorption interaction and surface properties of these The previous studies two main groups on different minerals, respectively [18,20,21]. Furthermore, the factors of the pelletizing performance, such as the pH value of solution and the metal cations, have also been discussed in detail [22,23]. However, MHA containing a high amount of organic matter generates a small amount of CO and/or H_2_ during the roasting process, which has an influence on pellet oxidation and consolidation [15]. To avoid these negative effects, a novel composite pellet binder using humic acid to modify bentonite, namely humic-acid-modified bentonite (HAMB), was developed in the laboratory. Furthermore, the interaction mechanism between humic substance-based binders and mineral particles has been discussed in detail in previous studies [24], which indicated that a humic substance-based could be a promising binder. However, the research on the effect of humic substance-based binders on green pellets properties, especially during the drying process, is lacking.

In general, the quality of the pellets was largely determined by the induration reaction during the process of preheating and roasting, which started only after the drying process of green pellet was finished [25]. The drying process of green pellets of iron ore concentrate is an intermediate step in the preparation of green pellets and preheating and roasting of pellets, which is usually realized by convective heat transfer with hot air. Setting the pellet drying stage can prevent the green pellets from cracking and pulverizing in the preheating stage, which would make the whole pellet layer less permeable and eventually affect the yield and quality of the pellets. This makes the drying process crucial in the industrial production of pellets, especially in the grate-rotary kiln process [26,27]. Therefore, research on the green pellet properties of a typical magnetite concentrate and three binders (bentonite, MHA, HAMB) and their performance during the drying process were investigated in this study. Based on experimental data, the relationship between moisture content in the pellets and compression strength during the drying process was determined. Additionally, the comprehensive effect and thermal behavior of humic substance on green pellets was investigated by FTIR and TG-DSC analysis.

## 2. Materials and Methods

### 2.1. Materials

#### 2.1.1. Magnetite Concentrate

In the test, the magnetite concentrate for pelleting, whose main chemical composition is shown in Table 1, was provided by a Chinese pelletizing plant. It can be seen that the contents of Total Fe and SiO_2_ were 67.25% and 5.60%, respectively, while the contents of other elements were very low. Table 2 shows the particle size distribution and other properties of the magnetite concentrate. The particle size distribution was measured by hydraulic sieving method, and the values of particle size distribution were 0.18% (+74 μm), 6.47% (−74~+38 μm), 93.35% (−38 μm), respectively. Additionally, the value of specific surface area was obtained by Blaine method (National standard GB/T 8074–2008).

#### 2.1.2. Binders

There were three types of binders (bentonite, MHA and HAMB) applied to the preparation of green pellets. Table 3 shows the main chemical compositions of the bentonite, which was also supplied by the same pelleting plant. The MHA binder was obtained from lignite, whose main organic elements and proximate analysis was reported in the previous article [24]. Additionally, previous research described the detailed method for preparing the HAMB, and its main chemical composition is shown in Table 4 [24].

### 2.2. Methods

#### 2.2.1. Pelletizing Tests

The mixture, consisting of 5 kg magnetite concentrate and a defined dosage of binder (1.0% of bentonite, 0.5% of MHA and 0.5% of HAMB, respectively), was put in a Φ 1000 mm disc pelletizer with an edge of 200 mm and an inclination angle of 45° to prepare green pellets. The total time of the balling process was 12 min, and the set rotation speed was 25 r/min. The diameter and moisture content of green pellets were controlled manually at 12–13 mm and 9.0% ± 0.5%, respectively. Then, 20 pellets from each batch were taken to detect properties of green pellets, such as drop numbers and compression strength, and the average value was recorded.

#### 2.2.2. Batch Drying Experiments

**Figure 1:**
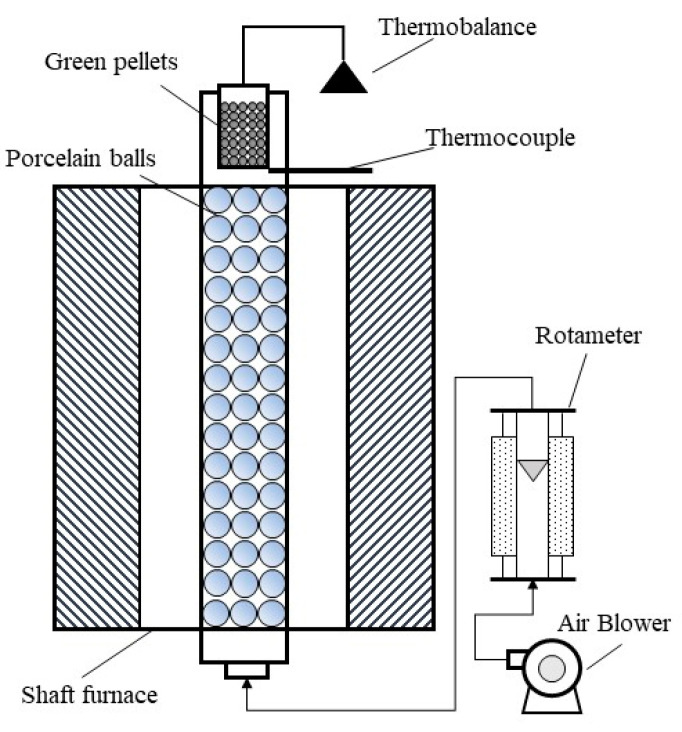
Schematic figure of the self-equipped device for drying test.

A Φ 650 × 1000 mm vertical tube furnace was modified, which was used to conduct the drying experiment. The schematic figure of the self-equipped device is shown in Figure 1. Under the condition of the set wind temperature and flow rate, the corresponding bed height of green pellets was placed into the pot, and then the mass varying with time during the drying process was recorded by the thermobalance. The series of drying rate was calculated using Equation (1), and the drying rate curve was plotted.

(1) $$\mathrm{DR}=\frac{\mathrm{dw}}{∆t}$$

where DR is the drying rate (%/min), dw is the water content change in the pellets(wt.%), g; and Δt is the corresponding time (min).

Additionally, the cracking temperature of green pellets was also measured with this device.

#### 2.2.3. FTIR and TG-DSC Analysis

As the previous studies indicated, the humic substance contained lots of functional groups, which can be active and interact with mineral particle. Therefore, the Fourier transform infrared spectra (FTIR, Thermo Nicolet Nexus 670 FTIR spectrometer, Gaithersburg, MD, USA) was applied to characterize the functional groups of the MHA in the range of 400–4000 cm^−1^.

The decomposition, dehydroxylation and/or decarboxylation of binder might affect the quality of pellets during the drying process. Therefore, it was necessary to study the thermal stability of binder by the TG-DSC technology (NETZSCH STA 449 C, Selb, Bavaria, Germany).

## 3. Results and Discussion

### 3.1. Effect of Different Binders on Green Pellets Quality

As shown in Table 5, which highlights the properties of prepared green pellets with three binders (1.0% of bentonite, 0.5% of MHA and 0.5% of HAMB, respectively), the properties of green pellets containing bentonite seemed to be the best, especially at the wet strength and cracking temperature. However, the dosage of bentonite was twice that of humic substance-based binders, whose index of dropping number was not better than 0.5% HAMB, and the other indices had no significant difference. Meanwhile the dropping number values of 0.5% HMA were lower than the production requirement 3.0 (Times·0.5 m^−1^). Therefore, the effect of 0.5% HAMB on green pellets was the best.

### 3.2. Drying Dynamics of Green Pellets

Due to the importance of the drying process, especially in the grate-rotary kiln process, many studies on pellet drying mechanisms and mathematical simulation models have been discussed in detail [25,28,29,30,31,32]. Therefore, the main purpose of this study was to apply these theories and models to analyze the effect of humic substance-based binders on green pellets during the drying process.

#### 3.2.1. Green Pellets with Bentonite

**Figure 2:**
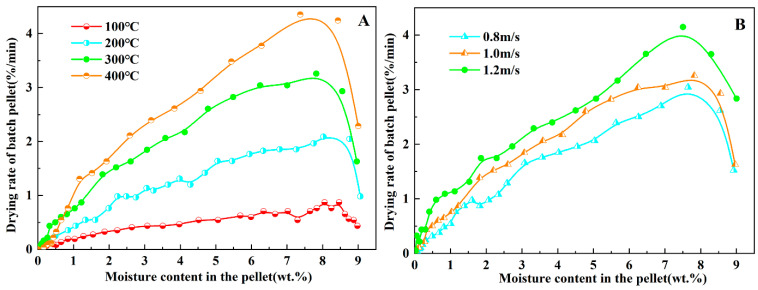
Drying characteristic curve of different wind temperature (**A**) and wind flow rate (**B**) on green pellets containing bentonite.

The drying of green pellets is usually accomplished by convective heat transfer of hot air. The hot air temperature became one of the main influencing factors of the drying process. Increasing the drying wind temperature can increase the quantity of heat supplied for drying in the unit time. It can significantly decrease the complete drying time and increase the drying rate. As the lowest cracking temperature of three binders was 413 °C, the drying medium temperatures were set at 100 °C, 200 °C, 300 °C and 400 °C. The corresponding drying wind flow rate and bed height were 1.0 m/s and 100 mm, respectively. The drying characteristic curves of green pellets containing bentonite at different wind temperatures are shown in Figure 2A.

It can be seen that the drying rate curve significantly varied from 100 °C to 300 °C with the wind temperature. However, compared to the drying rate of 300 °C, there was no significant increase in the drying rate curve of 400 °C. As the drying rate was controlled by evaporation and water/vapor inner diffusion, when the temperature was above 200 °C, the increase in drying rate slowed down [28]. In order to avoid the thermal cracking of green pellets from occurring during the drying process, a temperature of 300 °C was chosen as the wind temperature of subsequent tests.

Wind flow rate was another important factor in the drying process, whose effect was essentially similar to the wind temperature. Increasing the wind flow rate can decrease the drying time, which was caused by the vapor pressure difference between the inside and surface of the green ball. Figure 2B shows the drying characteristic curves of green pellets containing bentonite under different wind flow rates. Under the same drying temperature (300 °C) and the bed height (100 mm), a higher wind flow rate can make the water vapor diffuse to the pellet surface and air faster. Although the effect of wind flow rate was not as significant as that of wind temperature, choosing a wind flow rate of 1.2 m/s as the setting parameter can shorten the drying time.

#### 3.2.2. Green Pellets with Humic Substance-Based Binders

**Figure 3:**
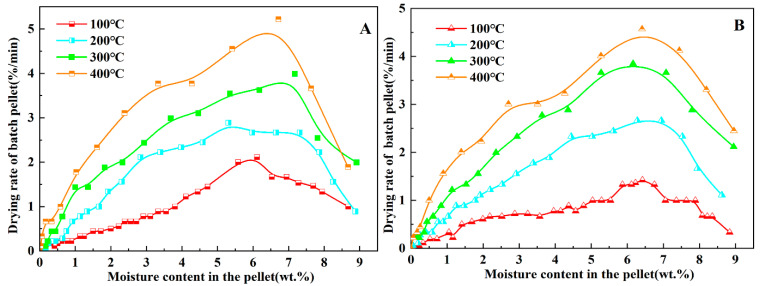
Drying characteristic curves of (**A**) MHA and (**B**) HAMB at different wind temperatures.

Considering that the effect of wind flow rate on green pellets containing bentonite was not as significant as that of wind temperature. While the thermal cracking of green pellets is the main influencing factor in the drying process, it is closely related to the drying medium temperature of green pellets. Therefore, the effect of wind temperature on green pellets containing humic substance-based binders should be mainly studied during the drying process. The corresponding drying characteristic curves of different wind temperature with humic substance binders are depicted in Figure 3, with the setting of the drying medium flow rate (1.2 m/s) and bed height (100 mm).

As shown in Figure 3B, the drying characteristic curves of the green pellets containing HAMB, and the green pellets containing bentonite at different wind temperatures, showed similar trends: the drying rate varied significantly with wind temperatures between 100 °C and 300 °C. However, there was no significant increase in the drying rate when the wind temperature increased to 400 °C. Figure 3A shows the drying characteristic curves of HMA-containing green pellets, which highlighted a distinctly different trend of the drying characteristics curve in the temperature range from 100 °C to 400 °C compared to the drying curve of the green pellets containing HAMB. Meanwhile, the drying rate of green pellets containing HMA at a low temperature (100 °C) was faster than that of green pellets containing HAMB. It can be inferred that the wind temperature had a big influence on a single humic substance-based binder.

**Figure 4:**
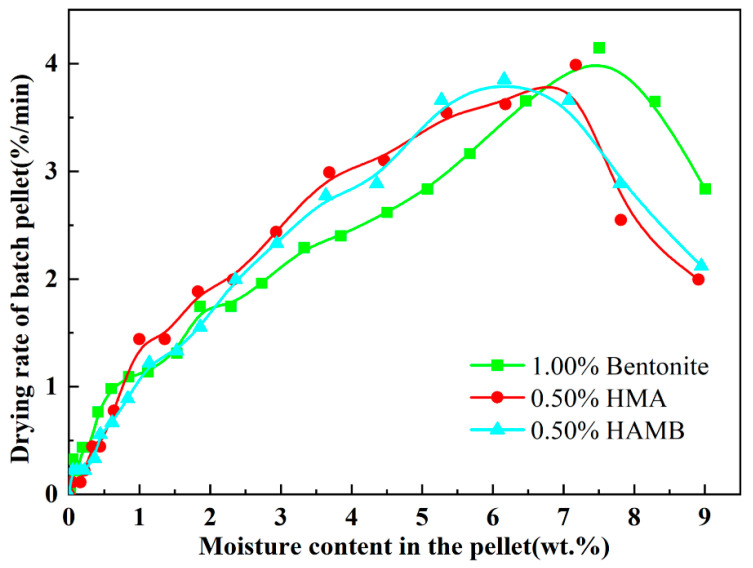
Drying characteristic curve of green pellets with different binders under same drying conditions.

In practice, the thermal cracking of green pellets generally occurred at the drying process of decelerated stage (or second drying stage), which was caused by the pellet mechanical strength not being able to endure the inner vapor pressure of green pellets at that time [28]. Furthermore, Fan et al. [27] demonstrated that the apparent activation energy (Ea) of the decelerated stage can comprehensively represent the drying resistance of water evaporation and/or vapor diffusion and indicate the thermal cracking performance of green pellets. Based on the Arrhenius law, the apparent activation energy (Ea) of the decelerated stage can be gained by the rate constants of the decelerated drying stage, which can be estimated by calculating the fitted slope of the corresponding drying curves within a certain range of moisture content [32]. Therefore, under the same conditions of wind temperature (300 °C), drying medium flow rate (1.2 m/s) and bed height (100 mm), the corresponding drying characteristic curves of three binders are depicted in Figure 4.

As shown in Figure 4, there were no significant differences between the drying characteristic curves of three binders at decelerated drying stage, and the actual cracking temperature of three binders (Table 5) were also similar. Therefore, a low dose of humic substance binder can achieve the same effect as a high dose of bentonite binder during the drying process.

### 3.3. Variations in Strength of Green Pellets during Drying Process

**Figure 5:**
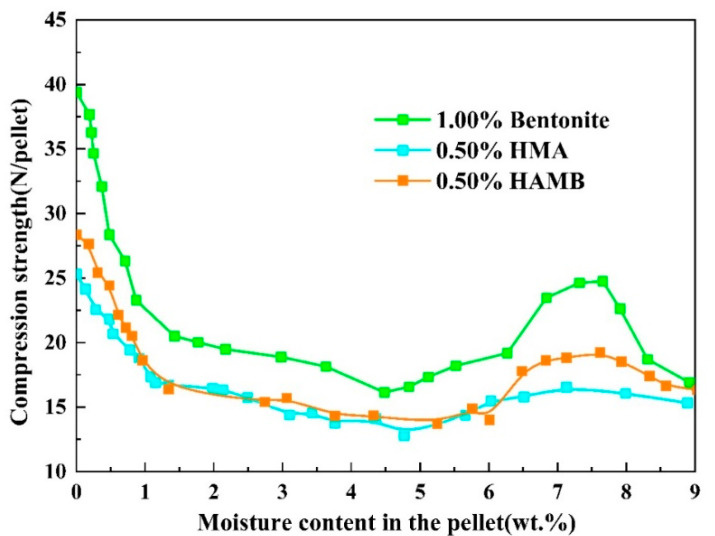
The relationship between moisture content in the pellet and compression strength.

It is well-known that the main function of the binder is to bond mineral particles together by controlling the moisture, affecting capillary forces, viscous forces and interparticle friction. Therefore, a “successful” binder can improve the pelletizing process and increase the strength of green pellets by slowing the migration of moisture from the interior to the surface of green pellets and increasing the viscosity of moisture in the green pellets [1,5]. Based on experiment data, the variations in the strength of green pellets with different types and corresponding doses of binders (1.00% bentonite, 0.50% HMA and 0.50% HAMB) during the drying process are plotted in Figure 5.

After the pelletizing process, the wet strength of the green pellet can reach a certain value due to the capillary force to bond mineral particles together into a mass. Then, at the beginning of the drying process, the capillary water in green pellets began to be eliminated, and the capillary in green balls began to shrink, which resulted in strengthening the capillary force and the adhesion between particles [1,5,28]. The strength of the green pellets gradually improved during this process. When most of the capillary water was removed, the capillary water that was left at particle contact points, which was named contact state capillary water. At this moment, the viscous forces caused by liquid bridges were largest, and the strength of pellets also reached the maximum [28,33]. As shown in Figure 5, the maximum value of the wet compression strength of the green pellets containing 1.00% bentonite was higher than that of the pellets containing 0.50% humic substance-based binders, and the compressive strength of the pellets containing 0.50% HMA was the lowest.

As the moisture in pellets continued to evaporate, the complete evaporation of capillary water led to the loss of capillary force, causing the strength of the particles to begin to decrease. Meanwhile, the viscous forces could hold mineral particles together as evaporation front penetrated towards the core of pellets. As the particles moved closer together, the increased friction forces between the particles improved the strength of the pellets. Toward the end of drying, the dry strength of pellets could reach a higher level due to the mechanical bridges and films that were formed by bentonite and a humic substance adhered to and/or concentrated at the particle contact points [5,28,33].

### 3.4. Thermal Performance of Binders

#### 3.4.1. FTIR Analysis of the MHA

FTIR spectroscopy is widely used to distinguish the bonding, chemical composition and structural changes in organic matter [34], and previous studies showed that a humic substance can adsorb on the surface of iron ore particles due to the polar functionality groups (such as -COOR, -COOH and -OH) [11,35]. In this paper, the effect of heating time and heating temperature on the structure of MHA during the drying process were discussed by FTIR spectroscopy technique, respectively.

**Figure 6:**
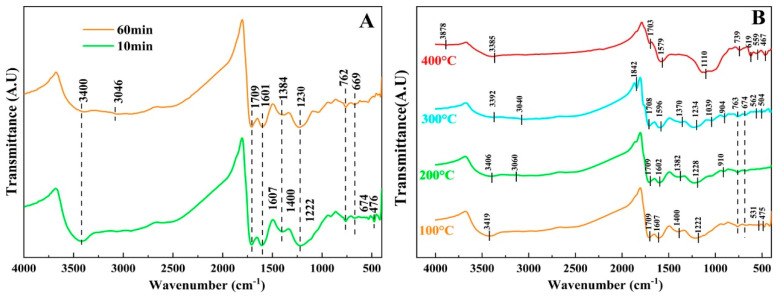
FTIR analysis of MHA at different heating times (**A**) and heating temperatures (**B**).

As is shown in Figure 6A, when the heating temperature was set at 100 °C, the FTIR spectra of the MHA with different heating time (10 min and 60 min) illustrated that the absorption peaks at 3419 cm^−1^ and 3400 cm^−1^ were attributed to the stretching vibration of -OH in carboxyl group linked by hydrogen bonding [34,36,37], and the weak peak around 3046 cm^−1^ during 60 min of heating was assigned to the -C=C- vibration in benzene ring [36,37]. The relative amount of functional groups can be reflected by the relative absorption peak intensities. Aside from the -OH content after 10 min of heating, which was higher than that for 60 min of heating, there was no obvious change in the structure of MHA with different heating times, indicating that heating time had little effect on MHA at a low temperature.

The heating time was set to 10 min, and the FTIR spectra of MHA with different heating temperatures is shown in Figure 6B. As shown in Figure 6B, the absorption peaks of the stretching vibration of -OH in the carboxyl group were at 3419 cm^−1^, 3406 cm^−1^, 3392 cm^−1^ and 3385 cm^−1,^ with the corresponding heating temperatures, respectively. This indicated that the content of -OH in the carboxyl group decreased when the heating temperature rose. Additionally, absorption peaks at 1400 cm^−1^, 1382 cm^−1^ and 1370 cm^−1^ were attributed to the stretching vibration of -OH in the phenolic groups [36,37], which disappeared at a heating temperature of 400 °C. In addition, the very weak absorption peak at 3878 cm^−1^ of 400 °C emerged, which was considered to be the stretching vibration of -OH linked by a metal element [36,37]. Above all, when the temperature was above 300 °C, the structural differences in MHA was significant.

Meanwhile, the contents of the polar functional groups (e.g., carboxyl and phenolic hydroxyl groups) in humic acids treated with different heating temperatures were also examined, as shown in Table 6.

It can be seen that the effects of heating temperature on the contents of the carboxyl group and phenolic hydroxyl group showed a tendency to decrease, when the heating temperature rose from 25 °C to 400 °C. When the heating temperature was between 25 °C and 100 °C, there was a strong decarboxylation reaction in the MHA which caused the content of carboxyl group to sharply decrease; the content of phenolic hydroxyl group also plummeted due to the dehydroxylation reaction. Then, the contents of these two polar functionality groups gradually declined from 100 °C to 400 °C.

The above results indicate that both the decarboxylation and dehydroxylation of humic acid can occur at a lower temperature (below 100 °C). Therefore, the drying temperature of humic acid binder could not be too high; otherwise, it would cause the failure of the MHA binder, which resulted from decreasing the content of polar functionality groups and/or destroying the structure of organic molecules.

#### 3.4.2. TG-DSC Analysis

**Figure 7:**
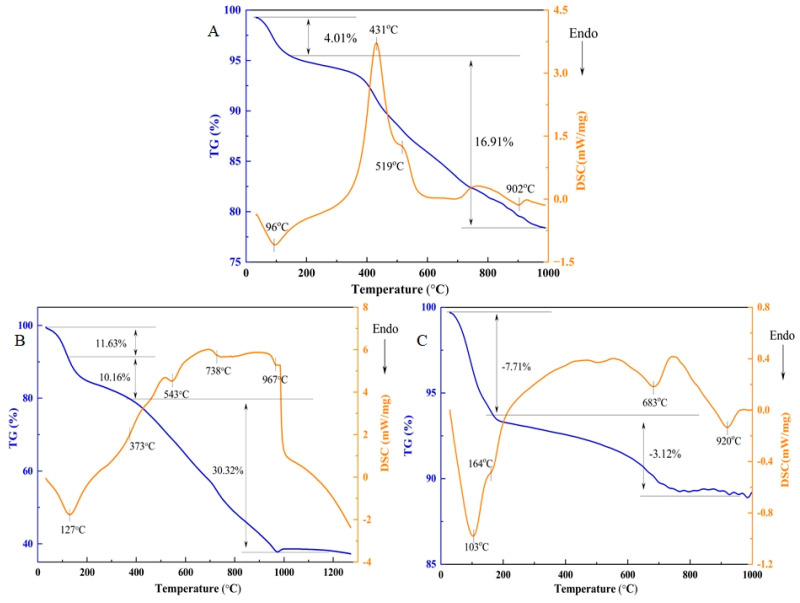
TG-DSC results of (**A**) HAMB, (**B**) MHA, and (**C**) bentonite.

As the novel composite pellet binder (HAMB) consisted of humic acid and bentonite, it was necessary to investigate the thermal behavior of these three binders. The TG-DSC analysis of three binders is shown in Figure 7. It can be seen from Figure 7B that there was 11.63% weight loss at the start of the TG curve of MHA when the temperature reached 127 °C, which was caused by the evaporation and dehydration of adsorbed and surface water in the MHA. Then, a small amount the MHA binder underwent reactions of decarboxylation, dehydroxylatio and/or decomposition with a temperature ranging from 127 °C to 373 °C. As the temperature continued to increase from 373 °C to 982 °C, a large number of MHA binders underwent a continuous intense thermal decomposition, decarboxylation, decarboxylation and/or dehydroxylation and combustion reactions. The corresponding weight loss of these two stages were 11.63% and 30.32%, respectively. When the temperature reached up to 1000 °C, the reactions of MHA were essentially complete and the weight of the residual was approximately 40%.

Combined with the TG-DSC analysis curve of bentonite and HAMB binders shown in Figure 7C and Figure 7A [38], respectively. The weight loss of 7.7% in Figure 7C was attributed to the dehydration of interparticle water, adsorbed water (endothermic reactions at 103 °C) and interlayer water (endothermic reactions at 164 °C) in the bentonite. Compared with TG-DSC of HAMB, as shown in Figure 7A, it was clear that the endothermic peak at 96 °C was the dehydration of interparticle water and adsorbed water in the HAMB with a weight loss of 4.01%. There was also no endothermic peak of dehydration of interlayer water. It was mainly because the humic acid reacted with the sites of bentonite adsorbed water and destroyed the interlayer structure, which made the endothermic peak of interlayer water disappeared. In addition, the weight loss of 3.12% was the result of the decomposition and combustion reactions in bentonite binder, which was much lower than that of HAMB binder. The results indicate that the HAMB binder was not a simple mix of humic acid and bentonite, which could be used to replace the bentonite to produce high-quality pellets.

## 4. Conclusions

A “successful” binder should improve the pelletizing process and hold the particles together during the drying process until pellets can be sent to the steel-making industry. In order to overcome the shortcomings of contaminants in bentonite, a humic substance-based binder was introduced to replace bentonite due to the reduction in pellet silica content.

The drying tests showed that a low dose of humic substance-based binder can achieve the same effect as a high dose of bentonite binder. An FTIR analysis and TG-DSC analysis found that both decarboxylation and dehydroxylation of humic acid can occur at lower temperatures (below 100 °C). Therefore, the drying temperature of green pellets containing MHA binder should not be too high.

Although the dropping number values of green pellets containing MHA did not meet the production requirement, the properties of green pellets with HAMB were better than those of green pellets containing bentonite. Meanwhile, the TG-DSC analysis indicated that the HAMB binder was not a simple mix of humic acid and bentonite. The humic substance could change the bentonite structure and reduce the thermal stability of bentonite, it could also decrease the silica content in the fired pellets when the HAMB was applied as the alternative binder.

## Figures and Tables

**Table 1:** Main chemical composition of magnetite concentrate (wt.%).

Total Fe	SiO_2_	Al_2_O_3_	CaO	MgO	P	K_2_O	Na_2_O	TiO_2_	LOI
67.25	5.60	0.14	0.13	0.23	0.01	0.02	0.09	0.12	−2.25

**Table 2:** Particle size distribution and other properties of magnetite.

Particle Size Distribution (%)			Balling Index	Specific Surface Area (cm^2^/g)
+74 μm	−74~+38 μm	−38 μm		
0.18	6.47	93.35	0.38	1971

**Table 3:** Main chemical compositions of bentonite (wt.%).

Total Fe	SiO_2_	Al_2_O_3_	CaO	MgO	K_2_O	Na_2_O	LOI
5.67	49.43	12.93	4.19	1.89	1.59	2.609	18.71

**Table 4:** Main chemical compositions of HAMB (wt.%).

Fe_2_O_3_	Al_2_O_3_	SiO_2_	TiO_2_	CaO	MgO	K_2_O	Na_2_O	LOI
2.29	16.37	57.63	0.16	3.63	4.45	0.31	0.06	15.01

**Table 5:** Properties of prepared green pellets.

Binder Type	Pellet Moisture	Dropping Number	Wet Strength	Cracking Temperature
Units	%	Times·0.5 m^−1^	N·Pellet^−1^	°C
1.00% bentonite	8.87	3.8	16.91	425
0.50% HMA	8.99	2.3	15.32	413
0.50% HAMB	9.03	4.3	16.34	421

**Table 6:** Effects of heating temperature on content of chemical groups in humic acid.

Chemical Groups (mmol/g)	25 °C	100 °C	200 °C	300 °C	400 °C
Carboxyl group	0.232	0.044	0.022	0.007	0.005
Phenolic hydroxyl group	4.668	1.326	1.248	1.08	0.87

## Data Availability

The data presented in this study are available on request from the corresponding author.
